# Current insights into the oncogenic roles of lncRNA LINC00355

**DOI:** 10.1002/cai2.91

**Published:** 2023-09-14

**Authors:** Jinze Shen, Xinming Su, Ming Pan, Zehua Wang, Yufei Ke, Qurui Wang, Jingyin Dong, Shiwei Duan

**Affiliations:** ^1^ Key Laboratory of Novel Targets and Drug Study for Neural Repair of Zhejiang Province, School of Medicine Hangzhou City University Hangzhou Zhejiang China

**Keywords:** ceRNA, chemotherapy resistance, lncRNA, LINC00355, miRNA, prognosis

## Abstract

Long noncoding RNAs (lncRNAs) are a class of nonprotein‐coding transcripts that are longer than 200 nucleotides. LINC00355 is a lncRNA located on chromosome 13q21.31 and is consistently upregulated in various cancers. It regulates the expression of downstream genes at both transcriptional and posttranscriptional levels, including eight microRNAs (miR‐15a‐5p, miR‐34b‐5p, miR‐424‐5p, miR‐1225, miR‐217‐5p, miR‐6777‐3p, miR‐195, and miR‐466) and three protein‐coding genes (*ITGA2*, *RAD18*, and *UBE3C*). LINC00355 plays a role in regulating various biological processes such as cell cycle progression, proliferation, apoptosis, epithelial‐mesenchymal transition, invasion, and metastasis of cancer cells. It is involved in the regulation of the Wnt/β‐catenin signaling pathway and p53 signaling pathway. Upregulation of LINC00355 has been identified as a high‐risk factor in cancer patients and its increased expression is associated with poorer overall survival, recurrence‐free survival, and disease‐free survival. LINC00355 upregulation has been linked to several unfavorable clinical characteristics, including advanced tumor node metastasis and World Health Organization stages, reduced Karnofsky Performance Scale scores, increased tumor size, greater depth of invasion, and more extensive lymph node metastasis. LINC00355 induces chemotherapy resistance in cancer cells by regulating five downstream genes, namely *HMGA2*, *ABCB1*, *ITGA2*, *WNT10B*, and *CCNE1* genes. In summary, LINC00355 is a potential oncogene with great potential as a diagnostic marker and therapeutic target for cancer.

## Graphical abstract

LINC00355 is a newly discovered long noncoding RNA that promotes tumor growth. This work overviews LINC00355's role in cancer through five aspects: its abnormal expression, regulatory mechanisms, biological functions, clinical significance, and resistance to chemotherapy. LINC00355 has the potential to serve as a new biomarker and therapeutic target for cancer treatment.

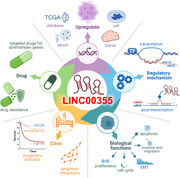

**Abbreviations**
BCa: bladder cancer
ceRNA: competitive endogenous RNA
CRC: colorectal cancer
EMCA: endometrial cancer
EMT: epithelial‐mesenchymal transition
EOC: epithelial ovarian cancer
GBM: glioblastoma
GC: gastric cancer
HCC: hepatocellular carcinoma
LGG: low‐grade glioma
lncRNA: long noncoding RNA
LUAD: lung adenocarcinoma
LUSC: lung squamous cell carcinoma
miRNA: microRNA
OVCA: ovarian cancer
PCGs: protein‐coding genes

## 1 INTRODUCTION

Long noncoding RNAs (lncRNAs) are a class of nonprotein‐coding transcripts longer than 200 nucleotides that are predominantly found in the nucleus [1]. Nuclear lncRNAs play a significant role in the transcriptional and posttranscriptional regulation of gene expression [2]. LncRNAs often participate in lncRNA/microRNA (miRNA)/messenger RNA (mRNA) networks as competitive endogenous RNAs (ceRNAs) [3, 4, 5]. Recent studies have discovered that dysregulation of lncRNAs can result in transcription‐replication conflicts and R‐loop‐mediated DNA damage [6].

LINC00355 is one of the most well‐studied lncRNAs and is located on human chromosome 13q21.31. Its expression is markedly upregulated in at least eight types of cancer, and thus LINC00355 has great potential as a cancer biomarker [7, 8, 9, 10, 11, 12, 13, 14]. Upregulation of LINC00355 is associated with high cancer risk factors, including clinical features, and poor prognosis of patients. LINC00355 regulates gene expression by interacting with chromatin and histones [7]. It promotes cancer cell proliferation, invasion, apoptosis, and other biological functions by targeting eight miRNAs. LINC00355 is involved in the regulation of the Wnt/β‐catenin signaling pathway [8, 9, 15] and the p53 signaling pathway [16]. LINC00355 also promotes the expression of three proteins by recruiting transcription factors [16, 17].

Recent studies have shown that LINC00355 plays an important role in tumorigenesis and tumor progression. This work describes the molecular mechanism and clinical significance of the abnormal expression of LINC00355 in cancer and provides insights for subsequent research directions on the role of LINC00355 in the occurrence and development of cancer.

## 2 LINC00355 IS MARKEDLY UPREGULATED IN VARIOUS CANCERS

LINC00355 expression was found to be upregulated in cell lines of eight different types of cancer compared with control cell lines (Table 1). These cancers include glioblastoma (GBM)/low‐grade glioma (LGG) [10], lung adenocarcinoma (LUAD) [11], lung squamous cell carcinoma (LUSC) [7], hepatocellular carcinoma (HCC) [8], gastric cancer (GC) [9, 16], colorectal cancer (CRC) [12, 17], bladder cancer (BCa) [13], and prostate cancer [14]. LINC00355 was also found to be markedly upregulated in seven types of cancer tissues compared with normal tissues, namely GBM/LGG [10], LUAD [11], LUSC [7], HCC [8, 15], GC [9, 16], CRC [12], and BCa [13, 22]. Furthermore, the level of LINC00355 in serum exosomes from BCa patients was found to be markedly higher than that of healthy individuals and patients with noncancer diseases [18, 19, 20, 21].

**Table 1:** Differential expression of LINC00355 in tumor cells, tissues, and exosomes.

Cancer	Expression	Level	Normal control	Tumor	References
BCa	Upregulated	Cell	SV‐HUC‐1	HT‐1197, HT‐1376, UM‐UC‐3, TCCSUP, and VMCUB1	[13]
	Upregulated	Exosome	NFs‐exosome of T24 and 5367	CAFs‐exosome of T24 and 5637	[18]
	Upregulated	Exosome	NFs‐exosome of paracancerous tissues from BCa patients	CAFs‐exosome of tumor tissues from BCa patients	[19]
	Upregulated	Exosome	NFs‐exosome of paracancerous tissues from BCa patients	CAFs‐exosome of tumor tissues from BCa patients	[18]
	Upregulated	Exosome	NFs‐exosome of paracancerous tissues from BCa patients	CAFs‐exosome of tumor tissues from BCa patients	[20]
	Upregulated	Exosome	Urine exosome of nonmalignant urinary disorders or normal urinary samples from 49 volunteers	Urine exosome of urinary samples from 59 BCa patients	[21]
	Upregulated	Tissue	Paracancerous tissues from 59 BCa patients	Tumor tissues from 59 BCa patients	[13]
CRC	Upregulated	Cell	CCD841CON	SW480, HT‐29, SW620, COLO205, HCT‐116, and T84	[17]
	Upregulated	Cell	FHC	SW480, HT‐29, and HCT‐116	[12]
	Upregulated	Tissue	Paracancerous tissues from 60 CRC patients	Tumor tissues from 60 CRC patients	[12]
GBM/LGG	Upregulated	Cell	NHAs	T98G, LN229, LN18, A172, and U251	[10]
	Upregulated	Tissue	Paracancerous tissues from 121 glioma patients	Tumor tissues from 121 glioma patients	[10]
GC	Upregulated	Cell	GES‐1	BGC‐823, MGC‐803, AGS, and SGC‐7901	[9]
	Upregulated	Cell	GES‐1	MGC‐803, BGC‐823, SGC‐7901, and HGC‐27	[16]
	Upregulated	Tissue	Paracancerous tissues from 48 GC patients	Tumor tissues from 48 GC patients	[9]
	Upregulated	Tissue	Paracancerous tissues from 72 GC patients	Tumor tissues from 72 GC patients	[16]
HCC	Upregulated	Cell	LO2	Bel‐7402, HepG2, Huh7, and Hep3B	[8]
	Upregulated	Tissue	Precancerous tissues from 10 HCC patients	Tumor tissues from 10 HCC patients	[15]
	Upregulated	Tissue	Paracancerous tissues from HCC patients	Tumor tissues from HCC patients	[8]
LUAD	Upregulated	Cell	HBE	A549, H1299, H292, H460, SPC‐A1, and LK2	[11]
	Upregulated	Tissue	Paracancerous tissues from 103 LUAD patients	Tumor tissues from 103 LUAD patients	[11]
LUSC	Upregulated	Cell	BEAS‐2B	SK‐MES‐1, NCI‐H226, A549, and NCI‐H2170	[7]
	Upregulated	Tissue	Paracancerous tissues from 30 lung SCC patients	Tumor tissues from 30 lung SCC patients	[7]
PC	Upregulated	Cell	WPMY1	PC3 and DU145	[14]

Abbreviations: BCa, bladder cancer; CAFs, cancer‐associated fibroblasts; CRC, colorectal cancer; GBM, glioblastoma; GC, gastric cancer; HCC, hepatocellular carcinoma; LGG, lower grade glioma; LUAD, lung adenocarcinoma; LUSC, lung squamous cell carcinoma; NFs, normal fibroblasts; PC, prostate cancer; SCC, squamous cell carcinoma.

To further investigate the differences in LINC00355 expression across various cancers, we conducted a pan‐cancer analysis using The Cancer Genome Atlas (TCGA) database and compared our findings with existing studies (see Supporting Information for details). Our analysis revealed that LINC00355 was markedly overexpressed in six types of cancer (ALL, AML, ESCA, LUAD, LUSC, and SKCM) in the TCGA database. The results for GBM/LGG [10], LUAD [11], and LUSC [7] were consistent with previous independent studies (Supporting Information: Table S1 and Figure S1).

## 3 MOLECULAR MECHANISMS OF LINC00355

**Figure 1:**
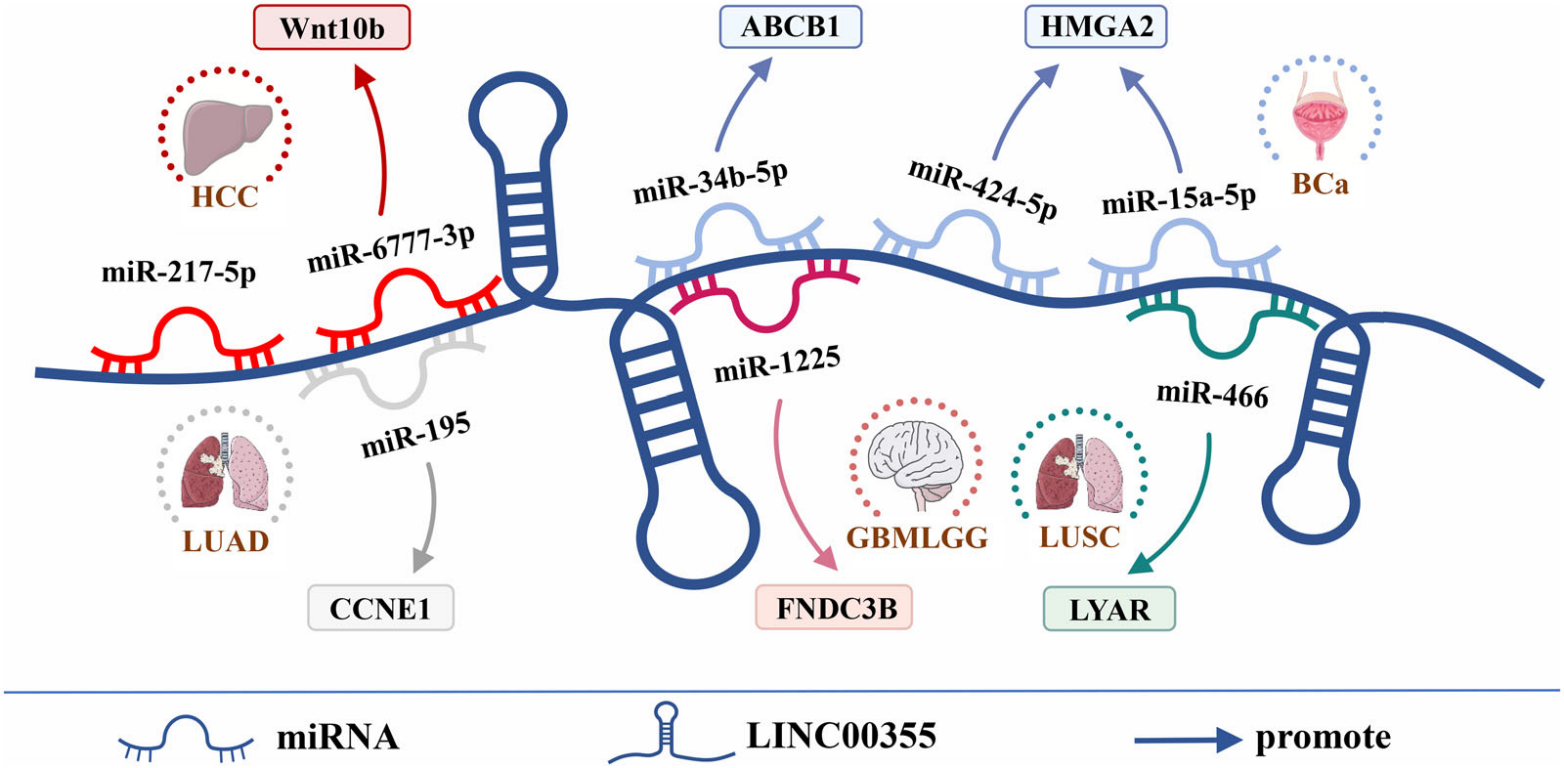
Regulatory mechanisms of LINC00355 at the posttranscriptional level. LINC00355 is involved in the posttranscriptional regulation of five cancers by targeting eight miRNAs. GBMLGG, glioblastoma low‐grade glioma; HCC, hepatocellular carcinoma; LUAD, lung adenocarcinoma; LUSC, lung squamous cell carcinoma; miRNA, microRNA.

LINC00355 plays a role in regulating gene expression at both the transcriptional and posttranscriptional levels (Table 2 and Figure 1). LINC00355 promotes the expression of *ITGA2*, *RAD18*, and *UBE3C* at the transcriptional level by recruiting transcription factors. At the posttranscriptional level, LINC00355 acts as a ceRNA to regulate eight downstream miRNA/mRNA axes to promote cancer progression.

**Table 2:** The ceRNA regulatory axes involved in LINC00355.

LINC00355/miRNA/PCG axis	Cancer	Binding site of LINC00355 and miRNA		Binding site of miRNA and PCG		References
		LINC00355 (5′–…–3′)	miRNA (3′–…–5′)	miRNA (5′–…–3′)	PCG (3′–…–5′)	
LINC00355/miR‐15a‐5p/HMGA2	BCa	UGCUGCUA	ACGACGAU	UAGCAGCA	AUCGUCGUA	[20]
LINC00355/miR‐34b‐5p/ABCB1		ACACUGCCUC	UGUGACGGAU	AGGCAGUGU	UCCGUCA	[18]
LINC00355/miR‐424‐5p/HMGA2		UCGUCGU	AGCAGCA	ACGACGAC	UGCUGCUA	[13]
		GUCGUCG	CAGCAGC	—	—	[13]
		UCGUCG	AGCAGC	—	—	[13]
LINC00355/miR‐1225/FNDC3B	GBM/LGG	GUACCCA	CAUGGGU	UGGGUAC	ACCCAUG	[10]
LINC00355/miR‐217‐5p	HCC	ATGCAGTA	UACGUCAU	—	—	[8]
LINC00355/miR‐6777‐3p/Wnt10b		GGtttagAGAGTGGA	CCcggucCUCUCACC	CCACUCU	UGAGACA	[15]
LINC00355/miR‐195/CCNE1	LUAD	GCCtcagccTCTGTGtaGCT	CGGttataaAGACACgaCGA	AGCAGCACaGaAauA	UCGUCGUaGcCcUcgU	[11]
LINC00355/miR‐466/LYAR	LUSC	TATGTGTA	AUACACAU	UACACAUacacgcAACA	AUGUGUAaaauauUUGU	[7]

Abbreviations: BCa, bladder cancer; ceRNA, competitive endogenous RNA; GBM, glioblastoma; HCC, hepatocellular carcinoma; LGG, lower grade glioma; LUAD, lung adenocarcinoma; LUSC, lung squamous cell carcinoma; miRNA, microRNA; PCG, protein‐coding gene.

### 3.1 LINC00355 regulates gene expression at the transcriptional level

LncRNAs have the ability to recruit transcription factors by forming R loops. They also directly interact with histone modification complexes, DNA‐binding proteins, and RNA polymerase II to act as promoters or inhibitors of transcription [23, 24]. For example, Linc‐Y1 recruits the transcription factor YY1 to activate the expression of multiple downstream genes [25]. In CRC, LINC00355 promotes *ITGA2* expression by recruiting GTF2B, a critical transcription factor involved in regulating various cancers [26, 27], thereby promoting cancer progression [17]. In GC, LINC00355 acts as a transcriptional activator that enhances the transcription of *RAD18* and *UBE3C*. This promotes the ubiquitination and degradation of the p53 tumor suppressor, leading to cancer progression [16].

The specific mechanism by which LINC00355 transcriptionally regulates *RAD18* and *UBE3C* has been unclear. To investigate this question, we predicted potential transcription factors that bind to the promoters or enhancers of *RAD18* and *UBE3C* genes (Figure 2). We screened the Signaling Pathways Project [28] and ChIP‐Atlas [29] databases to identify relevant transcription factors and found six potential transcription factors (Figure 2 and Supporting Information). Thus, LINC00355 may regulate the expression of three protein‐coding genes (PCGs) (*ITGA2*, *RAD18*, and *UBE3C*) in trans. Further experiments are needed to clarify the specific mechanisms of LINC00355 regulation of the six identified transcription factors.

Figure 2Investigating the recruitment of transcription factors by LINC00355 to regulate *RAD18* and *UBE3C*. (a) Identifying potential transcription factors for *RAD18* and *UBE3C*. We used database searches to identify potential transcription factors for *RAD18* and *UBE3C*. We then analyzed and visualized the related data. (b) Transcription factor retrieval and screening from SPP and ChIP‐Atlas databases. The upper histogram shows the size of the intersection between the two databases. The lower left histogram shows the size of each database's collection. The lower right dots and lines represent the overlap between the collections. (c) Enrichment of related transcription factors. We performed a log(fold enrichment) transformation on the binding scores and fold enrichments of relevant transcription factors in the two databases. A longer horizontal line indicates a higher degree of enrichment. (d) KEGG enrichment analysis of related transcription factors. We used the DAVID database (http://david.abcc.ncifcrf.gov) to perform the KEGG pathway analysis. A larger circle reflects a higher number of enriched genes. Our results showed that 6 out of 18 transcription factors, including CTBP1, MYC, LEF1, ARNT, NFE2L2, and RUNX1, were enriched into six pathways. (e) Potential binding regions of relevant transcription factors. The different colors on the chromosomes represent gene density, with red indicating high density and blue indicating low density. The horizontal lines of different colors in each row show the potential binding regions of different transcription factors on the chromosome where *RAD18* and *UBE3C* are located. (f) Motifs of related transcription factors. The figure displays the sequence logos of related transcription factors found in the JASPAR database (http://jaspar.genereg.net/). KEGG, Kyoto Encyclopedia of Genes and Genomes.

**Figure:**
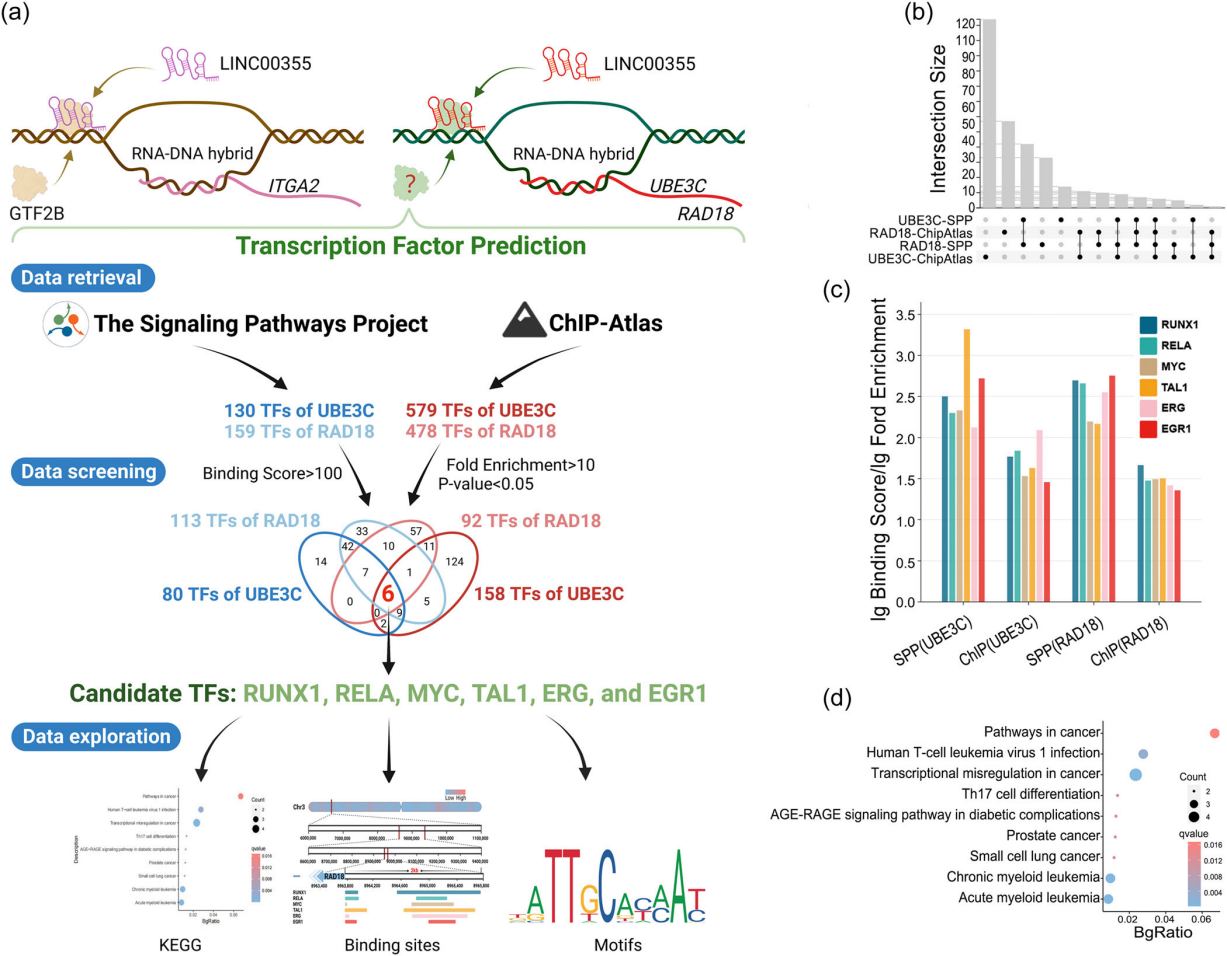

**Figure:**
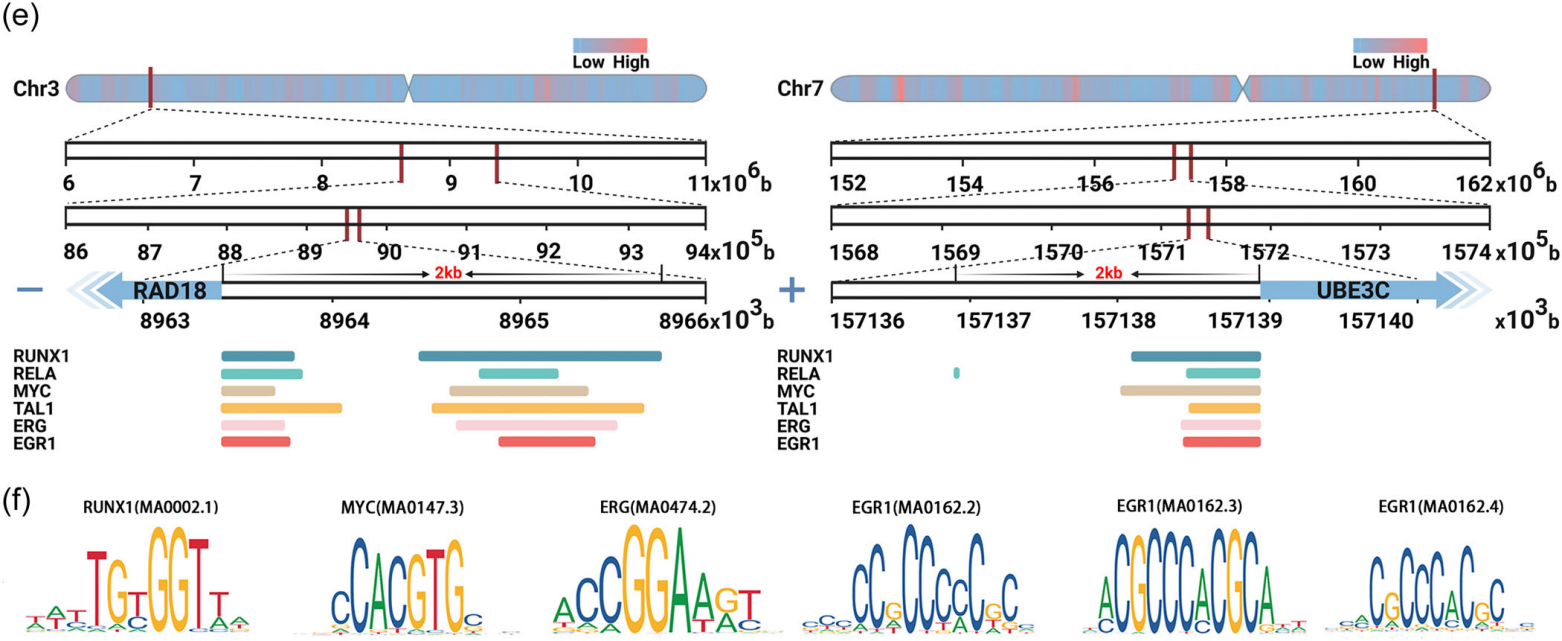

### 3.2 LINC00355 regulates gene expression at the posttranscriptional level

LncRNAs play a role in the posttranscriptional regulation of mRNAs through processes such as RNA editing, protein translation, and transport [30]. LncRNAs also act as ceRNAs to posttranscriptionally regulate gene expression through competition for shared miRNA binding sites [3]. Several studies showed that lncRNA/miRNA/mRNA axes are involved in regulating cancer progression [31, 32]. LINC00355 promotes the malignant phenotype of cancer cells through its function in eight ceRNA regulatory axes. These are the LINC00355/miR‐15a‐5p/*HMGA2* axis in BCa [20], the LINC00355/miR‐34b‐5p/*ABCB1* axis [18] and LINC00355/miR‐424‐5p/*HMGA2* axis [13] in GBM/LGG, the LINC00355/miR‐1225/*FNDC3B* axis [10] in HCC, the LINC00355/miR‐217‐5p axis [8] and LINC00355/miR‐6777‐3p/*WNT10B* axis [15] in HCC, the LINC00355/miR‐195/*CCNE1* axis [11] in LUAD, and the LINC00355/miR‐466/*LYAR* axis [7] in LUSC.

## 4 UPREGULATION OF LINC00355 PROMOTES THE MALIGNANT PHENOTYPE OF CANCER CELLS

**Figure 3:**
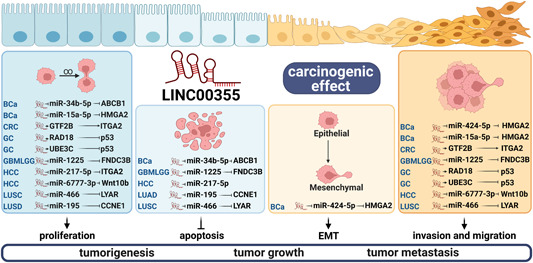
LINC00355 participates in the regulation of various biological behaviors of cells. LINC00355 regulates the biological behavior of various cancer cells through ceRNA networks or target genes. ceRNA, competitive endogenous RNA; EMT, epithelial‐mesenchymal transition.

LINC00355 plays a role in regulating biological behaviors such as proliferation, invasion, migration, and apoptosis of cancer cells (Table 3 and Figure 3). This was also shown to promote tumor progression in xenograft animal models.

**Table 3:** Biological functions of LINC00355 in vivo and in vitro.

Cancer	Regulatory mechanism	Pathway	Effect in vitro	Cell line	Effect in vivo	Xenograft model	References
BCa	—	—	Proliferation↑ and invasion↑	T24 and 5637	—	—	[19]
	LINC00355/miR‐15a‐5p/HMGA2	—	Proliferation↑ and invasion↑	T24 and 5637	—	—	[20]
	LINC00355/miR‐34b‐5p/ABCB1	—	Proliferation↑ and apoptosis↓	T24 and 5637	—	—	[18]
	LINC00355/miR‐424‐5p/HMGA2	—	EMT↑, invasion↑, and migration↑	T24, SW780, and HT‐1376	Tumor metastasis↑	HT‐1376 + BALB/C nude mice	[13]
CRC	LINC00355/GTF2B/ITGA2	—	Proliferation↑, invasion↑, and metastasis↑	COLO205	Tumor growth↑	COLO205 + BALB/c nude mice	[17]
GBM/LGG	LINC00355/miR‐1225/FNDC3B	—	Proliferation↑, colony formation↑, invasion↑, migration↑, metastasis↑, and apoptosis↓	U251 and T98G	—	—	[10]
GC	—	Wnt/β‐Catenin signaling	Cell cycle↑, proliferation↑, and apoptosis↓	AGS and SGC‐7901	—	—	[9]
GC	LINC00355/RAD18 and UBE3C	p53 Signaling	Proliferation↑, colony formation↑, invasion↑, and migration↑	BGC803, MGC803, and AGS	Tumorigenesis ↑	MGC803 or AGS + male NYG mice	[16]
HCC	LINC00355/miR‐217‐5p	Wnt/β‐catenin signaling	Proliferation↑ and apoptosis↓	Huh7 and Hep3B	—	—	[8]
	LINC00355/miR‐6777‐3p/Wnt10b	Wnt/β‐catenin signaling	Proliferation↑, invasion↑, and migration↑	HepG2, HepG2.2.15, and Huh7	Tumor growth↑	HepG2 + male BALB/c nude mice	[15]
LUAD	LINC00355/miR‐195/CCNE1	—	Cell cycle↑, proliferation↑, colony formation↑, and apoptosis↓	A549 and H1299	Tumor growth↑	A549 + male nude mice	[11]
LUSC	LINC00355/miR‐466/LYAR	—	Proliferation↑, invasion↑, migration↑, and apoptosis↓	NCI‐H2170	Tumor growth↑	NCI‐H2170 + male BALB/C nude mice	[7]

Abbreviations: BCa, bladder cancer; CRC, colorectal cancer; EMT, epithelial‐mesenchymal transition; GBM, glioblastoma; GC, gastric cancer; HCC, hepatocellular carcinoma; HNSCC, head, and neck squamous cell carcinoma; LGG, lower grade glioma; LUAD, lung adenocarcinoma; LUSC, lung squamous cell carcinoma.

### 4.1 LINC00355 promotes cell cycle progression

The cell cycle is a complex biological process that consists of G0, G1, S, G2, and M phases and is mainly regulated by cyclins and cyclin‐dependent kinases [33]. During the G1 phase, cells synthesize mRNA and protein in preparation for subsequent mitosis [34]. Cyclin E1 (encoded by the *CCNE1* gene) *CCNE1* is a highly conserved member of the cyclin family [35]. *CCNE1*Cyclin E1 interacts with CDK2 to form a complex that activates gene expression and DNA synthesis required for the S phase by phosphorylating target proteins [36]. The LINC00355/miR‐195/*CCNE1* mRNA axis was shown to inhibit G1 arrest and promote cell cycle progression in two LUAD cancer cell lines (A549 and H1299) [11].

### 4.2 LINC00355 promotes cell proliferation and xenograft tumor growth in vivo

Proliferation refers to the increase in cell numbers from cell division and is regulated by the activity of proteins related to the cell cycle [37]. Uncontrolled proliferation is a common characteristic of tumor cells [38]. LINC00355 was shown to promote cancer cell proliferation and tumor growth in xenograft animals by regulating the transcription of three genes (*ITGA2*, *RAD18*, and *UBE3C*) and participating in seven ceRNA axes.

In CRC, LINC00355 upregulates *ITGA2* expression by recruiting GTF2B. This promoted cell proliferation in the COLO205 cell line and tumor growth in xenografted BALB/c nude mice [17]. LINC00355 induced cell proliferation in three GC cell lines (BGC803, MGC803, and AGS) by promoting *RAD18* and *UBE3C* expression to mediate p53 ubiquitination. In vivo, studies showed that LINC00355 promoted tumor growth in male NYG mice xenografted with MGC803 or AGS cell lines [16].

*LYAR* is a nucleolar protein that plays a crucial role in cell proliferation and differentiation [39]. It increases the protein synthesis levels of cells by promoting ribosome biogenesis, thereby influencing the proliferation rate and function of cells [40]. LINC00355 was shown to promote the proliferation of LUSC cells (NCI‐H2170) by upregulating *LYAR* through the LINC00355/miR‐466/*LYAR* axis [7]. LINC00355 also regulates cell proliferation through six other ceRNA axes (Table 3). These are the LINC00355/miR‐15a‐5p/*HMGA2* axis [20] and LINC00355/miR‐34b‐5p/*ABCB1* axis [18] in BCa, the LINC00355/miR‐1225/*FNDC3B* axis in GBM/LGG [10], the LINC00355/miR‐217‐5p axis [8] and LINC00355/miR‐6777‐3p/*WNT10B* axis [15] in HCC, and the LINC00355/miR‐195/*CCNE1* axis in LUAD [11]. LINC00355 also promotes growth in xenograft mouse models through the LINC00355/miR‐6777‐3p/*WNT10B* axis [15] and LINC00355/miR‐466/*LYAR* axis [7] in HCC and LUSC.

### 4.3 LINC00355 inhibits the apoptosis of cancer cells

Apoptosis is a regulated cellular process that occurs under both physiological and pathological conditions. However, it is often inhibited during tumor development [41]. LINC00355 regulates apoptosis through four ceRNA axes (Table 3). These are the LINC00355/miR‐1225/FNDC3B axis in GBM/LGG [10], the LINC00355/miR‐217‐5p axis in HCC [8], the LINC00355/miR‐195/CCNE1 axis in LUAD [11], and the LINC00355/miR‐466/LYAR axis in LUSC [7].

### 4.4 LINC00355 promotes epithelial‐mesenchymal transition (EMT) of cancer cells

EMT is a process in which epithelial cells lose their characteristic features and acquire a mesenchymal phenotype [42]. This process can enhance the invasive ability of some cancer cells and lead to the generation of circulating tumor cells and cancer stem cells [43, 44]. LINC00355 was shown to upregulate the levels of EMT markers ZEB1 and vimentin and downregulate the level of E‐cadherin in three BCa cell lines (T24, SW780, and HT‐1376). *HMGA2* is a transcriptional regulator belonging to the high mobility group protein family [45]. It induces the expression of mesenchymal markers and reduces the secretion of epithelial markers by regulating multiple downstream pathways, such as Wnt/β‐catenin, TGF‐β, and Notch signaling pathways, thereby promoting EMT in cancer cells [46, 47]. LINC00355 was shown to upregulate *HMGA2* through the LINC00355/miR‐424‐5p/*HMGA2* axis and induce EMT in these cells [18].

### 4.5 LINC00355 promotes cancer cell invasion and migration

Migration and invasion are the processes by which malignant cells acquire active motility properties in tumors in situ and invade surrounding tissues [48]. These processes are key drivers of metastatic spread and often result in the formation of metastatic tumors [49]. LINC00355 was shown to promote invasion and migration in cancer cells and xenograft animals by regulating the transcription of three downstream genes (*ITGA2*, *RAD18*, and *UBE3C*) and participating in six ceRNA axes (Table 3).

The extracellular matrix (ECM) is a supportive and structural substance present between cells, and it inhibits cell invasion by acting as a physical barrier, signal transduction, and matrix stiffness [50]. Cell invasion requires the degradation and remodeling of the ECM to enable cells to spread from their original location to surrounding tissues or blood vessels [51]. *ITGA2*, an ECM receptor, is a transmembrane protein that promotes ECM remodeling and enhances tumor invasive potential [52]. In CRC, LINC00355 was shown to upregulate *ITGA2* expression by recruiting GTF2B, thereby promoting invasive ability in COLO205 cells [17]. In GC, LINC00355 induced the invasion and migration of three cancer cell lines (BGC803, MGC803, and AGS) by promoting the expression of *RAD18* and *UBE3C* to mediate the ubiquitination of p53 [16].

LINC00355 promotes the invasion and migration process of cancer cell lines through five ceRNA axes [20]. These are the LINC00355/miR‐15a‐5p/*HMGA2* axis and LINC00355/miR‐424‐5p/*HMGA2* axis in BCa [13], the LINC00355/miR‐1225/*FNDC3B* axis in GBM/LGG [10], the LINC00355/miR‐6777‐3p/*WNT10B* axis in HCC [15], and the LINC00355/miR‐466/*LYAR* axis in LUSC [7]. LINC00355 promoted tumor metastasis in vivo in a xenograft mouse model through the LINC00355/miR‐424‐5p/*HMGA2* axis in BCa [13].

### 4.6 LINC00355 is a high‐risk prognostic factor in cancer

LINC00355 is highly expressed in various cancers. High LINC00355 expression is associated with poor prognosis (overall survival, recurrence‐free survival, and disease‐free survival) and advanced pathological features in various cancers (Table 4).

**Table 4:** Association between LINC00355 and patient prognosis and pathological features.

Cancer	Sample size	Clinicopathological characteristics	Prognosis	References
BCa	59	High expression of LINC00355 is correlated to late TNM stage.	Poor OS and RFS	[13]
CRC	60	High expression of LINC00355 is correlated to big tumor size and more lymph node metastasis.	Poor OS	[12]
GBM/LGG	121	High expression of LINC00355 is correlated to low KPS and high WHO grade.	Poor OS and DFS	[10]
GC	72	High expression of LINC00355 is correlated to late TNM stage, high invasion depth, and more distant metastasis.	Poor OS	[16]
LUSC	30	—	Poor OS	[7]

Abbreviations: BCa, bladder cancer; CRC, colorectal cancer; DFS, disease‐free survival; GBM, glioblastoma; GC, gastric cancer; KPS, Karnofsky performance status; LGG, lower grade glioma; LUSC, lung squamous cell carcinoma; OS, overall survival; RFS, recurrence‐free survival; TNM, tumor node metastasis; WHO, World Health Organization.

High expression of LINC00355 predicts poor prognosis in five cancers (BCa, CRC, GBM/LGG, GC, and LUSC). This is reflected by a poorer overall survival in BCa [13], CRC [12], GC [16], GBM/LGG [10], and LUSC [7]; poorer disease‐free survival in GBM [10]; and poorer RFS in BCa [13]. The expression of LINC00355 also correlates with clinicopathological features. In BCa, high levels of LINC00355 correlate with advanced tumor node metastasis stages [13]. In CRC, high expression of LINC00355 is associated with larger tumor size and more lymph node metastases [12]. In GBM/LGG patients, high expression of LINC00355 is associated with lower Karnofsky Performance Status values and advanced World Health Organization stages [10]. In GC patients, high levels of LINC00355 are markedly associated with advanced TNM stage, deeper invasion depth, and more lymph node metastases [16].

## 5 LINC00355 PARTICIPATES IN THE REGULATION OF TWO CANCER‐RELATED SIGNALING PATHWAYS

**Figure 4:**
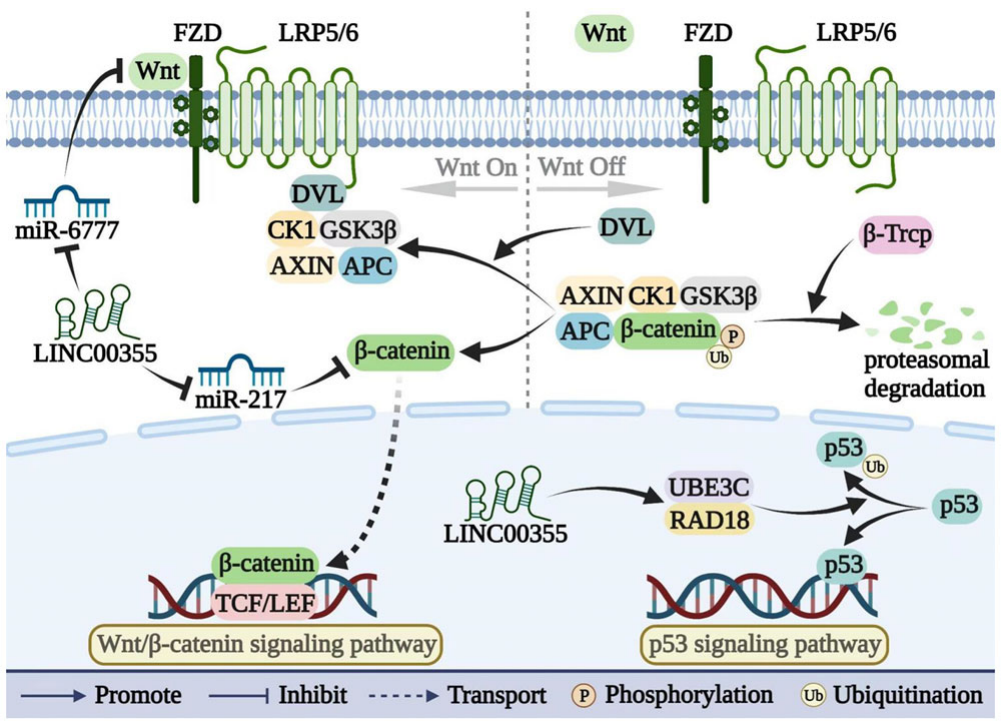
LINC00355 is involved in the regulation of the Wnt/β‐catenin and p53 signaling pathways. LINC00355 is involved in the regulation of the Wnt/β‐catenin and p53 signaling pathways.

LINC00355 plays a role in regulating two signaling pathways that promote cancer progression (Figure 4). LINC00355 is involved in the regulation of the Wnt/β‐catenin signaling pathway by targeting and suppressing miR‐217‐5p and miR‐6777‐3p. Additionally, LINC00355 promotes the transcription of *RAD18* and *UBE3C* to participate in the p53 signaling pathway.

### 5.1 LINC00355 regulates the Wnt/β‐catenin signaling pathway

The Wnt/β‐catenin signaling pathway is a highly conserved pathway that is typically repressed in adulthood and only reactivated during organ damage and regeneration [53]. Wnt signaling connects multiple signaling pathways and activates downstream effectors; germline mutations in the Wnt pathway can promote malignancy [54, 55]. *WNT10B* is a member of the Wnt family that participates in the activation of the Wnt/β‐catenin signaling pathway [56]. In HCC, LINC00355 upregulates the expression of *WNT10B* by inhibiting miR‐6777‐3p at the posttranscriptional level and it activates the Wnt/β‐catenin signaling pathway to promote malignant cell behavior [15]. LINC00355 also inhibits the Wnt/β‐catenin signaling pathway in HCC by sponging miR‐217‐5p, thus promoting cancer progression [8]. In GC, knocking down LINC00355 inhibits the Wnt/β‐catenin signaling pathway; however, the specific molecular mechanism has not yet been determined [9].

### 5.2 LINC00355 regulates the p53 signaling pathway

The p53 pathway responds to cellular stress, including oncogenic signals and DNA damage [57]. p53 is maintained at low levels and inactivated by its interaction with MDM2, which functions in part to mediate ubiquitination and degradation of p53 [58, 59, 60]. Ubiquitination is the process by which target proteins are specifically modified by ubiquitin molecules and targeted through the action of ubiquitin‐activating enzymes and ubiquitin‐regulating enzymes. This process plays a role in regulating inflammatory cell death and cancer [61, 62]. In GC, LINC00355 promotes the transcription of *RAD18* and *UBE3C* to mediate the ubiquitination and degradation of p53; this leads to the inhibition of the p53 signaling pathway and promotes the proliferation and invasion of GC cells [16].

## 6 LINC00355‐RELATED DRUGS AND POTENTIAL DRUG RESISTANCE

Chemotherapy is an important adjuvant in cancer treatment. However, patients often develop resistance to chemotherapeutic drugs, reducing their therapeutic effect [63]. lncRNAs regulate drug resistance‐associated gene expression through chromatin remodeling, transcriptional regulation, and posttranscriptional processing. These mechanisms can impact the effectiveness of chemotherapeutic drugs in cancer treatment [64].

By searching the CADDIE database (https://exbio.wzw.tum.de/caddie/) [65] and the DrugBank database (https://www.drugbank.ca) [66], we found several approved drugs that target LINC00355 downstream PCGs. The drugs include imatinib, palbociclib, and abemaciclib targeting *CCNE1*cyclin E1 and roxithromycin, saquinavir, amprenavir, reserpine, and mifepristone targeting *ABCB1*. Four PCGs downstream of LINC00355 have no approved targeted drugs in the CADDIE and DrugBank databases: *FNDC3B*, *HMGA2*, *LYAR*, and *WNT10B*.

ATP‐binding cassette (ABC) transporters are a family of proteins that use the energy from ATP hydrolysis to transport various molecules across the cellular membrane [67]. ABCB1, a member of the ABC family, is a transmembrane transporter that pumps drugs out of cells, reducing their effective concentration and increasing the drug resistance of cancer cells [68]. LINC00355 has been shown to upregulate ABCB1 through the LINC00355/miR‐34b‐5p/ABCB1 axis and induce cisplatin resistance in BCa cells (T24 and 5637) [18].

Studies have shown that some other LINC00355 downstream targets promote chemotherapy resistance in cancer. For example, *HMGA2* promotes cisplatin resistance in CRC and CC [69, 70] and oxaliplatin resistance in CRC [71]; *ABCB1* promotes erastin, docetaxel, and paclitaxel resistance in OC [72, 73]; *ITGA2* promotes paclitaxel resistance in OC [74]; *WNT10B* promotes doxorubicin resistance in TNBC [75] and cisplatin resistance in CRC [76]; and *CCNE1* promotes platinum resistance in epithelial ovarian cancer, ovarian cancer, and endometrial cancer [77, 78].

## 7 DISCUSSIONS

LncRNAs are noncoding RNAs that are involved in the regulation of almost every gene [79]. Differences in lncRNA expression can predict cancer patient prognosis [80], and marker lncRNAs can be used to predict patient response to treatment [81]. Systematic research has been conducted on the structure, localization, and regulation mechanism of lncRNAs [82, 83].

LINC00355 is located on chromosome 13q21.31 and has been consistently found to be highly expressed in various cancers, making it a promising biomarker in cancer diagnosis. However, the types of tumors studied so far have been limited. Furthermore, there is a lack of research on serum levels of LINC00355 in cancer, leaving the value of LINC00355 in assisting cancer diagnosis to be further explored. Future research should expand the range of cancer types studied and investigate the expression differences of LINC00355 in serum to assess its potential role in early cancer screening and noninvasive diagnosis. High expression of LINC00355 has been associated with advanced pathological stage and increased risk of poor prognosis. However, research on LINC00355 and prognosis has been limited by the narrow range of tumor types studied and the lack of follow‐up data. Future studies should expand the scope of cancer types and increase the number of follow‐up patients to determine the relationship between LINC00355 expression and patient survival.

LINC00355 recruits transcription factors or acts as a ceRNA in the regulatory network of cancer and promotes cancer through the Wnt/β‐catenin and p53 signaling pathways. While there have been preliminary studies on the regulatory mechanisms of LINC00355 in various tumors, most research has focused on its role as a ceRNA, and there is a lack of studies on other potential molecular mechanisms, such as molecular scaffolding. Future research should incorporate bioinformatics approaches to continue to explore the potential mechanisms of LINC00355 in cancer. Uncovering the regulatory mechanisms of LINC00355 in cancer may help clarify its potential for cancer diagnosis and therapy.

LncRNAs have been reported as drug targets for various diseases, and some lncRNA‐targeted drugs are in clinical trials [84]. For example, Andes‐1537 targets mitochondrial noncoding RNA and is in a phase I trial for solid tumors [85]. While no research is currently ongoing on drugs targeting LINC00355 or its downstream PCGs, the potential value of LINC0355 as a cancer therapeutic target is enormous. The molecular mechanisms linking LINC00355 and cancer cell resistance to chemotherapy, radiotherapy, and immunotherapy remain unknown. Further exploration of these mechanisms and the identification of effective drugs targeting LINC00355 and related targets is necessary, along with verification of adverse reactions and drug resistance using clinical trial data.

## 8 CONCLUSIONS

This work provides a summary of the expression and functions of LINC00355 in cancer and its prognostic value in cancer. It also describes the molecular basis and regulatory mechanism of the ceRNA networks involving LINC00355 and their roles in multiple biological functions in cancer. Our study highlighted the shortcomings of current research and speculated on LINC00355's potential role in chemotherapy and drug resistance. Given that LINC00355 consistently exhibits high expression in various cancers and its related regulatory mechanisms have been linked with promoting cancer, future research is required to better understand the molecular mechanisms of LINC00355. These findings will provide a solid theoretical foundation for the potential application of LINC00355 in clinical diagnosis and treatment.

## AUTHOR CONTRIBUTIONS

**Jinze Shen**: Data curation (equal); formal analysis (equal); methodology (equal); resources (equal); software (equal); writing—original draft (equal). **Xinming Su**: Data curation (equal); investigation (equal); validation (equal); visualization (equal); writing—original draft (equal). **Ming Pan**: Data curation (equal); visualization (equal); writing—original draft (equal). **Zehua Wang**: Investigation (equal); visualization (equal); writing—original draft (equal). **Yufei Ke**: Visualization (equal). **Qurui Wang**: Visualization (equal). **Jingyin Dong**: Project administration (equal); supervision (equal); writing—review and editing (equal). **Shiwei Duan**: Conceptualization (equal); funding acquisition (equal); supervision (equal); writing—review and editing (equal).

## CONFLICT OF INTEREST STATEMENT

The authors declare no conflict of interest.

## ETHICS STATEMENT

Not applicable.

## INFORMED CONSENT

Not applicable.

## Supporting information

Supporting information.

## Data Availability

All data generated or analyzed during this study are included in the article.
