# Supplementary material for: Current insights into the oncogenic roles of lncRNA LINC00355
Source: Cancer Innov. 2023 Sep 14;2(6):448–62. doi: 10.1002/cai2.91 (PMC10730005; doi:10.1002/cai2.91)
Supplement: Supplementary file 1 — Supporting information. [file CAI2-2-448-s001.docx]

**Current insights into the emerging oncogene LINC00355**

**Authors:** Jinze Shen^1,2,#^, Xinming Su^1,#^, Ming Pan^1^, Zehua Wang^1^, Yufei Ke^1^, Qurui Wang^1^, Jingyin Dong^1,2,*^, Shiwei Duan^1,2,*^

1. Department of Clinical Medici­ne, School of Medicine, Hangzhou City University, Hangzhou, Zhejiang, China

2. Key Laboratory of Novel Targets and Drug Study for Neural Repair of Zhejiang Province, School of Medicine, Hangzhou City University, Hangzhou, Zhejiang, China

#: JS and XS are co-first authors of this work.

*: Correspondence should be addressed to Drs. Shiwei Duan (duansw@zucc.edu.cn) and Jingyin Dong (dongjy@zucc.edu.cn)

**Supplementary Materials**

**TEXT**

Pan-cancer analysis of LINC00355

(Page 2)

Potential effects of LINC00355 on RAD18 and UBE3C transcription

(Page 2-3)

**Table S1.** TCGA data and existing studies of LINC00355

(Page 4)

**Table S2.** Chromosomal information, motifs, and experiments for related transcription factors

(Page 5)

**References**

(Page 6)

**Figure S1.** Expression of LINC00355 in TCGA pan-cancer database

(Page 7)

**Pan-cancer analysis of LINC00355**

We obtained LINC00355 expression data (TPM) for 32 tumors from the GDC-TCGA and GDC-TARGET databases (https://xenabrowser.net/) (1, 2). After performing a log2(TPM+0.001) transformation, we calculated the percentile rank of LINC00355 among all non-zero expressed lncRNAs in these 32 cancer types. Figure S1A shows that LINC00355 expression was very high in 6 tumors (>0.75 quantiles, Q4), high in 10 tumors (0.5-0.75 quantile, Q3), intermediate in 5 tumors (0.25-0.5 quantile, Q2), and low in 11 tumors (<0.25 quantile, Q1). Figure S1B displays the significance of the difference in LINC00355 expression between normal and tumor samples in each cancer type using the unpaired Wilcoxon test. TCGA data analysis revealed that LINC00355 was significantly overexpressed in 7 cancers (ALL, AML, ESCA, GBM/LGG, LUAD, LUSC, and SKCM) (adjusted P<0.05), with P values adjusted by Bonferroni for 32 cancer types. Table S1 shows that LINC00355 was significantly upregulated in 3 cancers (GBM/LGG, LUAD, and LUSC) according to TCGA data and previous research reports and has potential as a cancer diagnostic biomarker. Previous studies confirmed upregulation of LINC00355 expression in 5 cancers (BLCA, COAD, LIHC, PRAD, and STAD), but TCGA data analysis did not find significant overexpression of LINC00355 in these cancers. This may be due to fewer non-cancer control samples for these cancer types in TCGA (N<10, Table S1).

**Potential effects of LINC00355 on RAD18 and UBE3C transcription**

At the transcriptional level, LncRNA can regulate gene expression near its transcription site in cis or regulate gene expression far from its transcription site in trans (3). LINC00355 acts as a transcriptional activator in the nucleus to promote the expression of downstream proteins RAD18 and UBE3C, thereby promoting cancer progression (4). As shown in Figures 2A and 2B, using two databases, Signaling Pathways Project (SPP) (5) and ChIP-Atlas (6), we examined LINC00355's potential effect on RAD18 and UBE3C transcription. We obtained 80 and 113 transcription factors with strong binding ability to UBE3C and RAD18 respectively (binding score>100) from the SPP database. Additionally, ChIP-Atlas database analysis revealed 158 and 92 transcription factors enriched within 2 kb upstream of UBE3C or RAD18 genes, respectively (fold enrichment>10, P <0.05). After intersecting the screening data from both databases, we identified 6 transcription factors that may regulate UBE3C and RAD18: RUNX1, RELA, MYC, TAL1, ERG, and EGR1.

We then investigated the chromosomal information, KEGG enrichment analysis, potential binding sites, structures, and motif sources of the genes encoding the transcription factors shown in Figure2A. Table S2 shows that the loci of these 6 transcription factor genes are not adjacent to the loci of LINC00355, suggesting that LINC00355 is less likely to regulate these transcription factors in cis. We used the DAVID database (https://david.ncifcrf.gov/home.jsp) (7) to conduct KEGG pathway analysis on 6 potential transcription factors and found that 4 of them (RUNX1, RELA, MYC, and ERG) are enriched in 9 human diseases (including Transcriptional misregulation in cancer, Acute myeloid leukemia, Chronic myeloid leukemia, Human T-cell leukemia virus 1 infection, Small cell lung cancer, Prostate cancer, AGE-RAGE signaling pathway in diabetic complications, Th17 cell differentiation, and Pathways in cancer) as shown in Figure 2D.

As shown in Figure 2E, we identified the potential binding sites of six transcription factors on or around the RAD18 and UBE3C gene sequences. We obtained a total of 6 motifs supported by existing experimental evidence from the JASPAR database (http://jaspar.genereg.net/) (8) as shown in Table S2 and Figure 2F. It’s important to note that the interaction mechanism between these transcription factors and LINC00355 is still largely unknown and requires further validation through more detailed and rigorous experiments, such as gain and loss of function studies.

In summary, LINC00355 is likely to trans-regulate the promoters and enhancers of RAD18 and UBE3C through these six transcription factors (including RUNX1, RELA, MYC, TAL1, ERG, and EGR1), thereby affecting their transcription levels.

**Table S1. TCGA data and existing studies of LINC00355.**

| **TCGA cancer** | **Sample size** | **Biological information**  **analysis results** | **Results of existing studies** |
| --- | --- | --- | --- |
| ACC | T=18, N=2 | — | Not studied |
| ALL | T=42, N=21 | Up-regulated | Not studied |
| AML | T=21, N=107 | Up-regulated | Not studied |
| BLCA | T=272, N=5 | — | Up-regulated in BCa tissues and BCa cells (HT-1197, HT-1376, UM-UC-3, TCCSUP, and VMCUB1) (9) |
| BRCA | T=226, N=13 | — | Not studied |
| CESC | T=33 | — | Not studied |
| CHOL | T=5 | — | Not studied |
| COAD | T=64, N=6 | — | Up-regulated in CRC tissues and CRC cells (SW480, HT-29, SW620, COLO205, HCT-116, and T84) (10) |
| ESCA | T=103, N=19 | Up-regulated | Not studied |
| GBMLGG | T=19, N=31 | Up-regulated | Up-regulated in GBM/LGG tissues and GBM/LGG cells (T98G, LN229, LN18, A172, and U251) (11) |
| HNSC | T=220, N=5 | — | Not studied |
| KICH | T=4, N=2 | — | Not studied |
| KIRC | T=53, N=4 | — | Not studied |
| KIRP | T=20, N=1 | — | Not studied |
| LGG | T=30, N=30 | — | Not studied |
| LIHC | T=203, N=4 | — | Up-regulated in HCC tissues and HCC cells (Bel-7402, HepG2, Huh7, and Hep3B) (12) |
| LUAD | T=229, N=26 | Up-regulated | Up-regulated in NSCLC tissues and NSCLC cells (A549, H1299, H292, H460, SPC-A1, SK-MES-1, NCI-H226, A549, NCI-H2170, and LK2 ) (13, 14) |
| LUSC | T=338, N=21 | Up-regulated |  |
| NBL | T=54, N=12 | — | Not studied |
| OV | T=172, N=2 | — | Not studied |
| PAAD | T=18, N=8 | — | Not studied |
| PCPG | T=81, N=2 | — | Not studied |
| PRAD | T=58, N=9 | — | Up-regulated in PC cells (PC3 and DU145) (15) |
| READ | T=29 | — | Not studied |
| SARC | T=71, N=3 | — | Not studied |
| SKCM | T=298, N=12 | Up-regulated | Not studied |
| STAD | T=179, N=8 | — | Up-regulated in GC tissues and GC cells (BGC-823, MGC-803, AGS, HGC-27, and SGC-7901) (16) |
| TGCT | T=95, N=160 | — | Not studied |
| THCA | T=15, N=9 | — | Not studied |
| THYM | T=12, N=1 | — | Not studied |
| UCEC | T=43, N=1 | — | Not studied |
| UCS | T=20, N=1 | — | Not studied |

Please check GDC (https://gdc.cancer.gov/resources-tcga-users/tcga-code-tables/tcga-study-abbreviations) for the full name of the TCGA abbreviations.

**Table S2. Chromosomal information, motifs, and experiments for related transcription factors**

| **Official symbol** | **Chromosome** | **Gene location(Assembly)** | **Class** | **Matrix ID** | **Source** | **Validation** |
| --- | --- | --- | --- | --- | --- | --- |
| RUNX1 | 21 | 34,787,801-36,004,667(GRCh38.p14) | Runt domain factors | MA0002.1 | — | (17) |
| RELA | 11 | 65,653,601-65,663,857(GRCh38.p14) | — | — | — | — |
| MYC | 8 | 127,735,434-127,742,951(GRCh38.p14) | Basic helix-loop-helix factors (bHLH) | MA0147.3 | (18) | (19) |
| TAL1 | 4 | 47,216,290-47,232,335(GRCh38.p14) | Basic helix-loop-helix factors (bHLH) | MA0091.1 | — | (20) |
| ERG | 17 | 38,367,261-38,661,783(GRCh38.p14) | Tryptophan cluster factors | MA0474.2 | (21) | (22) |
| EGR1 | 5 | 138,465,479-138,469,303(GRCh38.p14) | C2H2 zinc finger factors | MA0162.2 | ENCODE | (23) |
|  |  |  | C2H2 zinc finger factors | MA0162.3 | (21) |  |
|  |  |  | C2H2 zinc finger factors | MA0162.4 | ReMap |  |

RUNX1, RUNX Family Transcription Factor 1; RELA, RELA proto-oncogene; MYC, MYC Proto-Oncogene; TAL1, TAL bHLH transcription factor 1; ERG, ETS transcription factor ERG; EGR1 early growth response 1.

**References**

1. Cancer Genome Atlas Research N, Weinstein JN, Collisson EA, Mills GB, Shaw KR, Ozenberger BA, et al. The Cancer Genome Atlas Pan-Cancer analysis project. Nat Genet. 2013;45(10):1113-20.

2. Goldman MJ, Craft B, Hastie M, Repecka K, McDade F, Kamath A, et al. Visualizing and interpreting cancer genomics data via the Xena platform. Nat Biotechnol. 2020;38(6):675-8.

3. Nojima T, Proudfoot NJ. Mechanisms of lncRNA biogenesis as revealed by nascent transcriptomics. Nat Rev Mol Cell Biol. 2022;23(6):389-406.

4. Zhao W, Jin Y, Wu P, Yang J, Chen Y, Yang Q, et al. LINC00355 induces gastric cancer proliferation and invasion through promoting ubiquitination of P53. Cell Death Discov. 2020;6(1):99.

5. Ochsner SA, Abraham D, Martin K, Ding W, McOwiti A, Kankanamge W, et al. The Signaling Pathways Project, an integrated 'omics knowledgebase for mammalian cellular signaling pathways. Sci Data. 2019;6(1):252.

6. Zou Z, Ohta T, Miura F, Oki S. ChIP-Atlas 2021 update: a data-mining suite for exploring epigenomic landscapes by fully integrating ChIP-seq, ATAC-seq and Bisulfite-seq data. Nucleic Acids Res. 2022;50(W1):W175-82.

7. Sherman BT, Hao M, Qiu J, Jiao X, Baseler MW, Lane HC, et al. DAVID: a web server for functional enrichment analysis and functional annotation of gene lists (2021 update). Nucleic Acids Res. 2022;50(W1):W216-21.

8. Castro-Mondragon JA, Riudavets-Puig R, Rauluseviciute I, Lemma RB, Turchi L, Blanc-Mathieu R, et al. JASPAR 2022: the 9th release of the open-access database of transcription factor binding profiles. Nucleic Acids Res. 2022;50(D1):D165-D73.

9. Li WJ, Li G, Liu ZW, Chen ZY, Pu R. LncRNA LINC00355 promotes EMT and metastasis of bladder cancer cells through the miR-424-5p/HMGA2 axis. Neoplasma. 2021;68(6):1225-35.

10. Ruan Z, Deng H, Liang M, Xu Z, Lai M, Ren H, et al. Overexpression of long non-coding RNA00355 enhances proliferation, chemotaxis, and metastasis in colon cancer via promoting GTF2B-mediated ITGA2. Transl Oncol. 2021;14(1):100947.

11. Qi ZY, Wang LL, Qu XL. lncRNA LINC00355 Acts as a Novel Biomarker and Promotes Glioma Biological Activities via the Regulation of miR-1225/FNDC3B. Dis Markers. 2021;2021:1683129.

12. Luo X, M AB, Yang G, Yan Z, Fu X, Lu P, et al. LINC00355 triggers malignant progression of hepatocellular carcinoma via the sponge effect on miR-217-5p with the involvement of the Wnt/β-catenin signaling. J buon. 2021;26(5):1964-9.

13. Liang Y, Rong X, Luo Y, Li P, Han Q, Wei L, et al. A novel long non-coding RNA LINC00355 promotes proliferation of lung adenocarcinoma cells by down-regulating miR-195 and up-regulating the expression of CCNE1. Cell Signal. 2020;66:109462.

14. Sun X, Wang G, Ding P, Li S. LINC00355 promoted the progression of lung squamous cell carcinoma through regulating the miR-466/LYAR axis. Braz J Med Biol Res. 2020;53(12):e9317.

15. Jiang T, Guo J, Hu Z, Zhao M, Gu Z, Miao S. Identification of Potential Prostate Cancer-Related Pseudogenes Based on Competitive Endogenous RNA Network Hypothesis. Med Sci Monit. 2018;24:4213-39.

16. Luan PB, Sun XM, Yao J. LINC00355 inhibits apoptosis and promotes proliferation of gastric cancer cells by regulating Wnt/β-catenin signaling pathway. Eur Rev Med Pharmacol Sci. 2020;24(16):8377-83.

17. Meyers S, Downing JR, Hiebert SW. Identification of AML-1 and the (8;21) translocation protein (AML-1/ETO) as sequence-specific DNA-binding proteins: the runt homology domain is required for DNA binding and protein-protein interactions. Mol Cell Biol. 1993;13(10):6336-45.

18. Cheneby J, Gheorghe M, Artufel M, Mathelier A, Ballester B. ReMap 2018: an updated atlas of regulatory regions from an integrative analysis of DNA-binding ChIP-seq experiments. Nucleic Acids Res. 2018;46(D1):D267-D75.

19. Chen X, Xu H, Yuan P, Fang F, Huss M, Vega VB, et al. Integration of external signaling pathways with the core transcriptional network in embryonic stem cells. Cell. 2008;133(6):1106-17.

20. Hsu HL, Huang L, Tsan JT, Funk W, Wright WE, Hu JS, et al. Preferred sequences for DNA recognition by the TAL1 helix-loop-helix proteins. Mol Cell Biol. 1994;14(2):1256-65.

21. Jolma A, Yan J, Whitington T, Toivonen J, Nitta KR, Rastas P, et al. DNA-binding specificities of human transcription factors. Cell. 2013;152(1-2):327-39.

22. Wei GH, Badis G, Berger MF, Kivioja T, Palin K, Enge M, et al. Genome-wide analysis of ETS-family DNA-binding in vitro and in vivo. EMBO J. 2010;29(13):2147-60.

23. Meng X, Brodsky MH, Wolfe SA. A bacterial one-hybrid system for determining the DNA-binding specificity of transcription factors. Nat Biotechnol. 2005;23(8):988-94.

**Figure S1. Expression of LINC00355 in TCGA pan-cancer database**


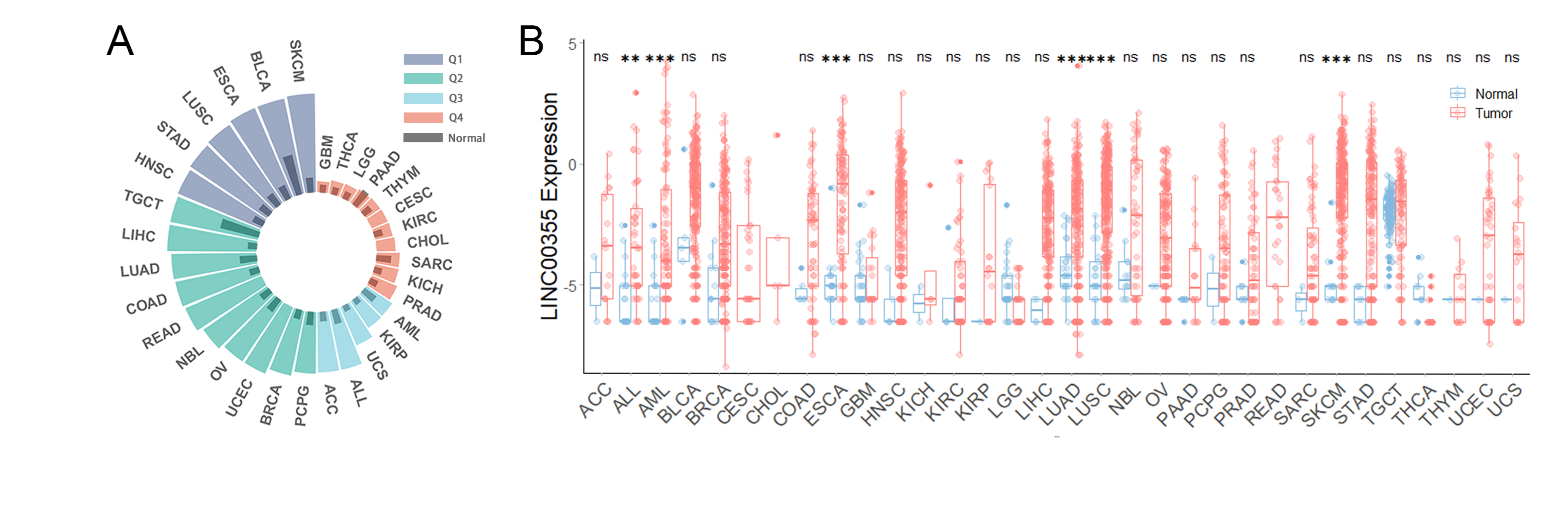


(A) LINC00355 Expression Levels in Pan-Cancer. LINC00355 was highly expressed in 6 tumors (>0.75 quantile, Q4), high in 10 tumors (0.5-0.75 quantile, Q3), moderately expressed in 5 tumors (0.25-0.5 quantile, Q2), and lowly expressed (<0.25 quantile, Q1) in 11 types of tumors (expression ranking/non-zero expression LncRNA number).

(B) LINC00355 Expression Significance in Pan-Cancer. "***" indicates adjusted p<0.001, "**" indicates adjusted p<0.01, "*" indicates adjusted p<0.05, and "ns" indicates no significant difference. LINC00355 was significantly overexpressed in 6 cancers (ALL, AML, ESCA, LUAD, LUSC, and SKCM).

Please check GDC (https://gdc.cancer.gov/resources-tcga-users/tcga-code-tables/tcga-study-abbreviations) for the full name of the TCGA abbreviations.
